# Small-scale resource tracking in a population of a long-lived insect

**DOI:** 10.1002/ece3.378

**Published:** 2012-09-24

**Authors:** Olof Widenfalk, Christer Solbreck, Hanna L Bergeå

**Affiliations:** Department of Ecology, Swedish University of Agricultural SciencesP.O. Box 7044, SE-750 07, Uppsala, Sweden

**Keywords:** Flowering pattern, prolonged diapause, resource exploitation, small scale patchiness, white swallow-wort

## Abstract

How plant-feeding insects distribute themselves and utilize their host plant resources is still poorly understood. Several processes may be involved, and their relative roles may vary with the spatial scale considered. Herein, we investigate small-scale patterns, namely how population density of a gall midge is affected by individual growth form, phenology, and microsite characteristics of its herb host. The long-lived plant individuals vary much with regard to number of shoots, flower abundance, and flowering phenology. This variation is connected to site characteristics, primarily the degree of sun exposure. The monophagous insect galls the flowers of the host plant – an easily defined food resource. It is a poor disperser, but very long-lived; diapausing larvae can stay in the soil for many years. Galls were censused on individual plants during 5 years; from a peak to a low in gall population density. Only a very small fraction of the flowers produced (<0.5%) were galled even in the peak year. Nevertheless, most plant individuals had galls at least 1 year. In a stepwise multiple regression, plant size (number of shoots) was found to be the most important predictor of gall density (galls/flower). However, gall density *decreased* more than one order of magnitude over the plant size range observed. There was also a weak effect of plant phenology. Early flowering plants had lower gall densities than those starting later. Sun exposure had no direct effect on gall density, but a path analysis revealed indirect effects via the timing of flowering. Gall population change was highly synchronous in different parts of the study area with no significant decrease in synchrony with distance.

## Introduction

The distribution of plant-feeding insects in the landscape is often affected by multiple processes, each of which may have a dominating effect on a different spatial scale (e.g., Menéndez and Thomas 2000; Krawchuk and Taylor 2003; Rabasa et al. 2005; Singer and Wee 2005; Roslin et al. 2006; Gripenberg and Roslin 2007). Many studies have been concerned with the factors determining insect occurrence or abundance in host plant patches on the landscape scale (Hanski and Gilpin 1991; Harrison 1991; Hanski 1994, 1997; Förare and Solbreck 1997; Thomas and Kunin 1999; Kéry et al. 2001). Relatively few have considered distributions on smaller scales (Heads and Lawton 1983; Doak 2000; Krawchuk and Taylor 2003; Cook and Holt 2006). For example, how do insects exploit the resources and microhabitats provided by individual herbaceous plants within the landscape-scale patches?

As a null model, insect distributions can be assumed to mirror the distribution of their food resources. However, several processes may cause deviations from such patterns, for example, risk spreading behavior among egg laying females (Root and Kareiva 1984; Förare and Solbreck 1997; Williams et al. 2001) or enemy interactions (Hopkins and Dixon 1997; Maron and Harrison 1997; Zeipel et al. 2006). Apparent deviations may also result from problems of defining the resources available to herbivorous insects (Dempster 1997, 1998). For example, differences in nutritional quality (Price 1991; Björkman 2000), host plant resistance (Fritz and Price 1988; Moran and Whitham 1990; Fernandes and Negreiros 2000), microclimatic conditions (Weiss et al. 1988; Solbreck 1995), and phenological synchronization (Hunter 1992; Quiring 1994; Fox et al. 1997; Kerslake and Hartley 1997; Ngakan and Yukawa 1997; Yukawa 2000) may render substantial parts of potentially available resources useless for herbivore populations.

Local insect densities are determined by a combination of inter-patch movements and within-patch population growth. The relative contributions of these processes vary much between different resource–exploiter systems (Andersson and Hambäck 2012). Whereas movement habits may dominate the dynamics of short-lived strong fliers utilizing temporally variable resources, in situ population growth is more likely to dominate the dynamics of long-lived, poor dispersers utilizing stable resources. This study is concerned with a system at the latter end of this spectrum, namely a poor disperser, with a very long life and population age structure, which utilizes a resource which varies little from year to year. Very little is known about the patterns of resource exploitation in this kind of system, particularly, so at the scale of individual plants and in a multi-year perspective.

Herein, we explore, in a 5-year study, how structural characteristics of the individual plant and of the plant site affect the distribution of a gall midge, *Contarinia vincetoxici* Kieffer galling the flowers of a long-lived perennial herb, White Swallow-wort, *Vincetoxicum hirundinaria* Medicus (L.). The insect is strictly monophagous on this plant, and the resource used (flowers) is well defined (Widenfalk et al. 2002). The larvae are stationary in the galls which are easy to find. This means that the abundance of both insects and their food resources are easily measured.

The host plant displays patchiness on two spatial scales. On the landscape scale, it is distributed in small (usually 1–500 m^2^) and fairly isolated patches (hundreds of meters to kilometers) (Ågren et al. 2008). There is also a small scale patchiness, which is the focus of this study. This patchiness is created by plant individuals, which form discrete tussocks. These tussocks display considerable variation in height (= shoot length), width (= number of shoots), and in density as well as in the abundance of flowers and in the temporal pattern of flowering. These structural characteristics of plants are in turn affected by microsite characteristics. Plant individuals grow in a heterogeneous microenvironment, ranging from sun-exposed rocky areas to shaded parts on often deeper soil. The degree of sun exposure has been shown to have a strong and dominating impact on plant growth form in *V. hirundinaria* (Ågren et al. 2008). However, microsite conditions may also have direct effects on the insects. For example, sun exposure is likely to affect the thermal environment of the insect during all phases of its life cycle. Hence, in addition to plant structural characteristics, we aim to disentangle possible direct and indirect effects of sun exposure on gall density.

The interaction between *V. hirundinaria* and *C. vincetoxici* is characterized by very local interactions, which are potentially of very long endurance. The plant is very long-lived and the insect combines a very long life cycle with poor dispersal. The short-lived adult midges have poor colonizing ability as in many cecidomyiids (Briggs and Latto 2000; Widenfalk et al. 2002). Larvae complete their development within the flower gall in about 2 weeks, and then the fullgrown larvae drop to ground beneath the plant. There they enter a prolonged diapause, which extends over several years, and which results in a “seed bank” of diapausing larvae. A cohort of larvae entering the soil in 1 year may emerge as adults over a period of more than 10 years (Solbreck and Widenfalk 2012). The kind of life history syndromes shown by the gall midge and its host plant suggests that very local patterns of population fluctuations may arise.

We first describe patterns of microsite and plant variation, to illustrate the wide variation in their characteristics. Based upon a 5-year census of galls, we analyze the degree of synchrony in gall population fluctuations over the study area. Then, we analyze the degree of resource exploitation by the insect population in relation to traits and microsite characteristics of individual plants. Five characteristics are considered, namely (1) the number of shoots per individual (genet size), (2) the number of flowers per shoot (ramet), (3) the number of flowers per individual (4) the timing of the start of flowering (phenology), and (5) the duration of the flowering period. Finally, using path analysis, we investigate possible direct and indirect effects of sun exposure.

## Material and Methods

**Figure 1:**
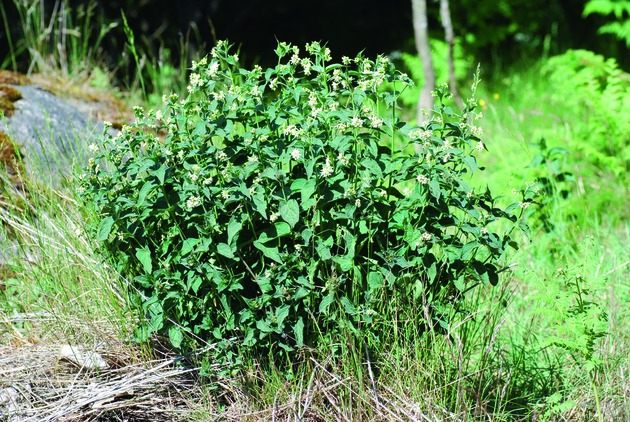
A *Vincetoxicum hirundinaria* individual during flowering. This plant individual consists of about 40–50 shoots.

The host plant *V. hirundinaria* is distributed throughout Eurasia with its north-western distribution limit in south-eastern Scandinavia (Donadille 1965; Hultén 1971; Hultén and Fries 1986). The clonal plant individuals consist of up to one hundred or more shoots (Fig. 1). Each shoot produces up to one hundred flowers during the extended flowering period, which normally begins in early June and lasts until late July or August. Plant individuals are very long-lived and their size, in terms of shoot and flower production changes little from year to year (Ågren et al. 2008; Solbreck unpubl.data).

Adult *C. vincetoxici* midges are small (about 2-mm long), fragile, and short-lived with a life span of at most a couple of days. Females oviposit in young flower buds of *V. hirundinaria,* which then develop into galls (Widenfalk et al. 2002). The gall is formed by the basally swollen and unopened petals. The gall is larger than an ordinary flower bud, and it usually has a reddish tint, thus being easily recognized. Each gall contains on average 15 midge larvae. They feed in the gall for about 2 weeks, whereafter they drop to the ground to over-winter in the soil in larval cocoons. The larvae enter a prolonged diapause of variable length, but with a median duration of at least 6 years (Widenfalk and Solbreck 2005; Solbreck and Widenfalk 2012). The adult females produce about 35 eggs, which means that they on average may induce two galls. Galls are found during a large part of the flowering period, from the beginning of June until the end of July (Widenfalk et al. 2002).

The study was conducted in a patch of *V. hirundinaria* at Bladåker (59^°^58′N, 18^°^17′E) 45 km north-east of Uppsala, Sweden. This is a fairly typical landscape scale patch of intermediate size, and with ordinary plant growth form and gall density for this part of Sweden. The patch is quite variable in terms of plant microenvironment, ranging from sun-exposed rocky ground to shaded, forested parts. Gall abundance was censused in the entire patch 1997–2003 by counting and marking (with a permanent marker pen on the nearest leaf) all new galls every week through the galling season, and calculating a yearly sum.

**Figure 2:**
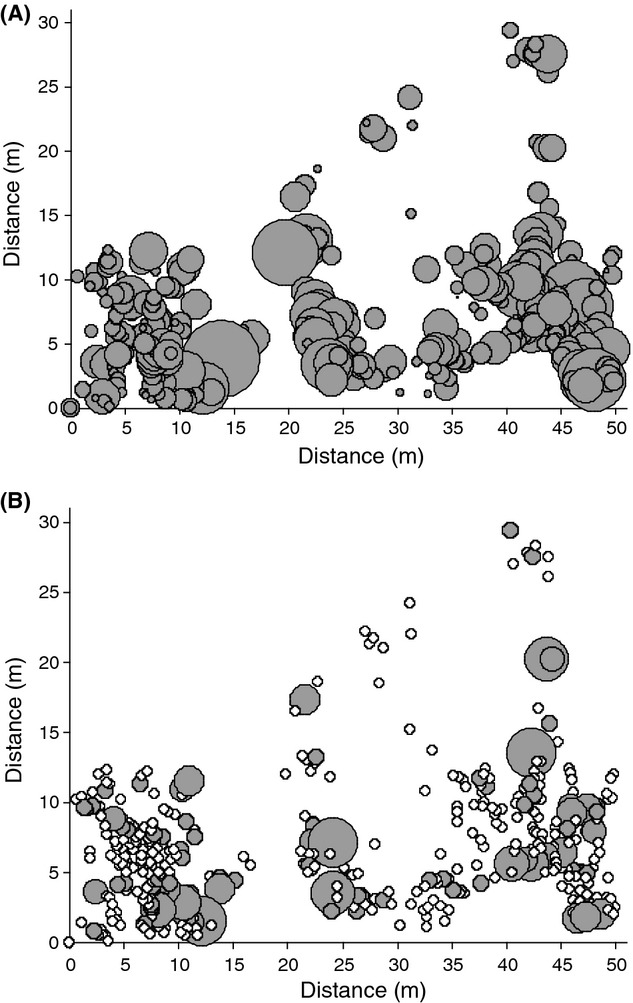
Schematic maps of the study site in 2001 with all 425 host plant individuals and their galls. (A) Plant size, expressed as the number of flowers (30–6,527), is proportional to circle area (cf Fig. 3C). (B) The abundance of galls. Open circles represent plant individuals without galls, and the area of the filled circles is proportional to the number of galls (0–8 per plant).

About 75% of the patch (30 × 50 m) formed the main study area, in which all galls were both mapped and counted 1998–2002 (Fig. 2). The mapping was more detailed in 2001. All 425 flowering plant individuals (covering a total surface area of approximately 50 m^2^) were identified and mapped and all galls mapped unto those plant individuals (Fig. 2). Using the maps of the distribution of galls between 1998–2000 and 2002 and the map of plant individuals (and galls) from 2001, a 5-year series of gall abundances for 230 of the host plant individuals could be established. This data set was used to analyze the role of individual plant characteristics and sun exposure on gall occurrence and density.

Plant individuals are usually easily distinguished on the basis of variation in flower color and morphology. Flowering phenology of each plant individual was monitored in 2001 on three randomly chosen marked shoots, which were checked weekly for the occurrence of opened flowers. The start of flowering was measured as the week, counted from the first of June, when at least one opened flower had appeared on any of the three shoots, and the length of the flowering period was measured as the number of weeks that any of the three shoots were flowering. (The different shoots of an individual plant are highly synchronized with regard to flowering.) Even though the start and the length of the flowering period will vary between years depending on the weather, the measure taken in 2001 is a good *relative* measure of the commencement and the length of the flowering period during the other years of study (C.S. unpubl. data).

The number of flowers per shoot was measured at the end of the flowering period in 2001 from counts of flower stalk scars. (Because flowers are numerous and flowering time very extended one can not count all flowers directly on the plant.) On each plant individual, the number of flowers was estimated on each of three randomly chosen shoots by counting (1) the number of flower stalk scars of the lowest node with flowers (*x*_1_) and (2) the number of flowering nodes (*x*_2_). The number of flowers per shoot (*y*) was estimated by the formula *y* = 5.512*x*_2_+1.9528*x*_1_, *R*^2^ = 0.931, df = 188 (Ågren et al. 2008). The mean number of flowers per shoot was then calculated from the three shoots and multiplied by the number of flowering shoots per plant to calculate the number of flowers per individual.

The number of shoots and flowers per shoot varies little from year to year. For example, the number of shoots (*S*) and flowers (*F*) on 29 individuals were compared in 2001 and 2002 and were found to be almost equal and strongly positively correlated (*S*_2002_ = 0.914*S*_2001_ + 0.384, *R*^2^ = 0.977, and *F*_2002_ = 1.031 *F*_2001_−18.13, *R*^2^ = 0.971). Therefore, we utilize the flower data from 2001 also for the other 4 years.

In the analysis, we maintain number of shoots and number of flowers per shoot as separate variables (rather than just calculating the number of flowers per individual). As plant individuals form rather distinct tufts they can be seen as tiny “islands” where island size is expressed by the number of shoots, whereas flowers per shoot can be seen as a resource density measure.

The degree of sun exposure of each individual plant was measured in the following way. A transparent plastic sheet was mounted vertically along the periphery of a horizontal semicircular plate and mounted on a tripod. The path of the sun at midsummer (as viewed from the circle center of the base plate) was delineated on the sheet and the sun′s position for each hour marked. The device was placed at the top of the plant and aligned with the aid of a compass and a spirit-level. Looking from the circle center the number of hours (between 6.00 and 18.00 hours) the plant was not shaded by vegetation could then be read. Because of the prolonged diapause of *C. vincetoxici* gall abundance in successive years does not (except to a small extent) represent successive generations, but rather members of a cohort emerging as adults over several years. We thus chose to use mean gall density per flower and year for the entire 5-year period as a response variable. It was log-transformed to achieve normality.

Effects on gall densities were analyzed using simple and stepwise linear regression, using the regression procedure in the SAS statistical software version 9.1. The variables no. of shoots, no. of flowers/shoot, and no. of flowers/individual were log-transformed to achieve normality. The criterion for variable entry and removal from the stepwise models was set to a significance level of 0.1.

To separate the direct and indirect effects of sun exposure and plant characteristics on gall densities, a path analysis was performed (Sokal and Rohlf 1995; Legendre and Legendre 1998). This analysis is based on the standardized coefficients from simple and multiple regressions relating the variables in the path diagram. If a variable is hypothesized to be affected by two other variables in a path diagram, the path coefficients are simply the standardized regression coefficients from a multiple linear regression with the two latter variables as independents. By comparing the path coefficient from a multiple regression with the coefficient from a simple regression (also standardized, i.e., correlation coefficients), one can obtain an estimate of the indirect and direct effects of the variable, respectively.

We hypothesized a model with both direct and indirect effects of microsite conditions (sun exposure) and only direct effects of plant characteristics. Path coefficients were obtained as the standardized regression coefficients from multiple and simple linear regressions using the regression procedure in SAS. Correlation coefficients, for studying causal and non-causal covariation, were obtained using the correlation procedure in SAS. Missing data were deleted listwise so that no individual with missing values in any of the variables were included in the analysis.

To overcome the problem that plants with zero galls are omitted from the analysis of the relationship between gall density and flowers per individual plant, we also conducted an analysis where we classified all plant individuals into size categories and calculated attack rates from the total number of galls and flowers in each category.

To analyze possible spatial autocorrelation patterns of gall density fluctuations yearly, gall data were pooled for each 5 × 5 m square of the study area (cf Fig 2B). This produced 14 time series with the criterion of a maximum of 3 zeroes (10 without zeroes, 2 with 1 zero, 1 with 2 zeroes and 1 with 3 zeroes). All time series were log(10) transformed (with zeroes replaced by 0.5). Distances between squares were measured between square centers and log(10) transformed.

The spatial autocorrelation in gall population change was analyzed using a Mantel-test (Legendre and Legendre 1998). The test consists of calculating the correlation of the matrix of pairwise correlations of population change and the matrix of distances. However, the significance cannot be directly assessed, because there are *N*(*N−*1)/2 entries for just *N* observations. The test is performed by permuting the matrices and calculating the same test statistic under each permutation and comparing the original test statistic to the distribution of test statistics from the permutations to generate a *P*-value. The number of permutations defines the precision with which the *P*-value can be calculated. We performed 9,999 permutations. The analysis was performed using the *R* statistical software.

## Results

### Variation in plant individuals and sun exposure

**Figure 3:**
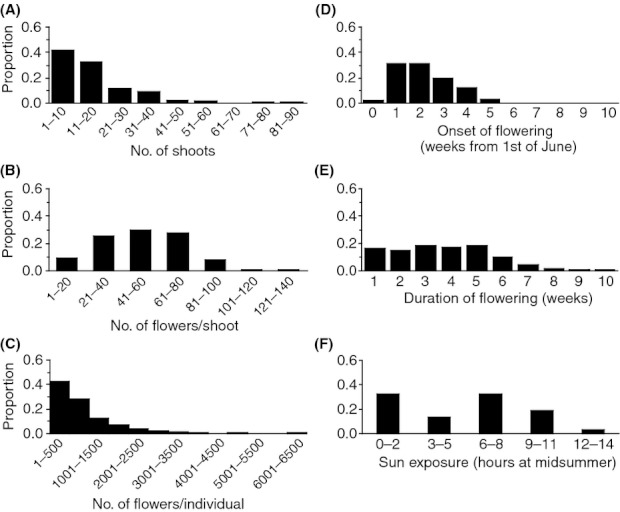
Frequency distribution of plant individuals with regard to (A) number of shoots, (B) number of flowers per shoot, (C) number of flowers/individual, (D) onset of flowering, (E) duration of flowering and (F) degree of sun exposure.

There was considerable variation between plant individuals with regard to size and floral production (Figs. 2A, 3). (but little change from year to year, see Material and methods). Plant size, expressed as number of shoots, varied from 1 to 85 with a strong skew toward small individuals (median 12) (Fig. 3A). The mean number of flowers per shoot showed a more normal distribution with a range from 8 to 139 (median 51) (Fig. 3B). The total number of flowers per plant ranged from 30 to 6,527 with a dominance of plant individuals with few flowers (median 610) (Fig. 3C).

There was also strong variation in the timing and duration of flowering. The first and the last individuals to initiate flowering differed by as much as 5 weeks, but more than 60% of the plants had initiated their flowering within 2 weeks of the appearance of the first opened flower (Fig. 3D). The duration of the individual flowering period varied from 1 to 10 weeks (Fig. 3E). The plant population was also variable with regard to sun exposure; from shade all day to sun exposure all day (Fig. 3F).

### Distribution of galls

**Figure 4:**
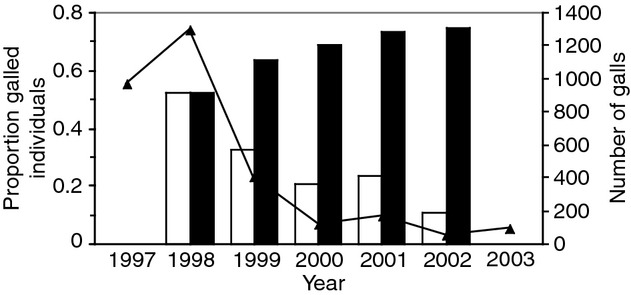
The proportion of galled plant individuals each year (open bars) and the cumulative proportion of galled individuals (black bars) for the 230 individually identified plants which were followed 1998–2002. Also shown (line with triangles) are changes in the total gall populations from 1997 to 2003.

The total gall population varied more than one order of magnitude during the period 1997–2003 (Fig. 4). Studies in neighboring areas indicate that densities were lower prior to 1997 at least back to 1995 (C.S. unpubl.data). Our data on gall distributions 1998–2002 (Fig. 4), hence represent the situation from a population peak to a population low.

The proportion of galled plant individuals ranged from 48% in the peak year 1998 to 11% during the population low in 2002. The cumulative proportion of galled plants increased every year despite the strong decrease in overall gall abundance, and most of the plant individuals (75%) had galls at least 1 year during 1998–2002 (Fig. 4).

Despite the high proportion of galled individuals, only a very small proportion of the total number of flowers was galled in any year. For example, of a total of approximately 200,000 flowers produced in 2001, only 0.08% were galled. This means (because flower abundance is fairly constant from year to year) that approximately 0.5% of the flowers were galled in the peak gall year.

**Figure 5:**
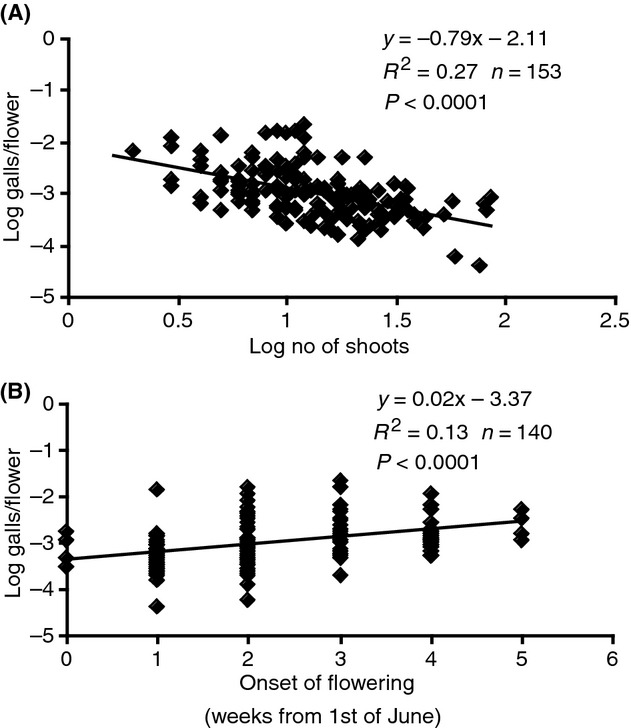
The relationship between the mean gall density (mean no of galls 1998–2002 for the plant individuals with at least 1 gall during the period), and the plant characteristics found significant in the multiple regression (Table 2). (A) number of shoots, (B) onset of flowering. Regression equations, *R*^2^-values as well as the number of individuals in the analysis (*n*), are indicated in the graphs.

Gall density varied with individual plant size. Plants with many shoots had much lower densities of galls/flower; mean gall density decreased by more than one order of magnitude over the size range observed (Table 1, Fig. 5A). The number of flowers per shoot had a small, but significant effect on individual gall density. There is also a significant effect of number of flowers per individual, which logically follows from the two previous relationships (Table 1).

**Table 1:** Results from linear regressions on the relationship between log mean number of galls per flower and all the assessed plant parameters in the study

Variable	Slope-parameter	*R*^2^	df	*P*
Log no. of shoots	−0.788	0.265	153	<0.0001
Log (no. of flowers/shoot)	−0.538	0.049	153	0.0062
Log (no. of flowers/individual)	−0.667	0.285	153	<0.0001
Onset of flowering	0.023	0.132	140	<0.0001
Duration of flowering	−0.032	0.017	153	0.1066
Sun exposure	−0.032	0.044	152	0.0098

There is a significant relationship between gall density and plant phenology; plants that started their flowering early in the season had lower gall densities (Table 1, Fig. 5B). Gall density is also lower on more sun-exposed plants (Table 1), but the duration of flowering did not significantly affect gall density (Table 1).

In a stepwise multiple regression (using all variables in Table 1 except number of flowers per individual which is calculated from the first two variables), the number of shoots per individual was the single most important variable explaining alone 26% of the variance in gall density (Table 2). The only other variable with a significant contribution was start of flowering, which explained an additional 7% of the variance (Table 2).

**Table 2:** Results from a stepwise linear regression. *R*^2^ = 0.33, *P* < 0.0001, *n* = 139. Variables not included in the model (*P* > 0.1): Log no of flowers/shoot, duration of flowering and sun exposure

Variable	Parameter estimate	Standard error	*F*	*P*
Intercept	−2.49	0.158	248	<0.0001
Log no. of shoots	−0.68	0.108	40	<0.0001
Onset of flowering	0.02	0.005	14	0.0003

**Figure 6:**
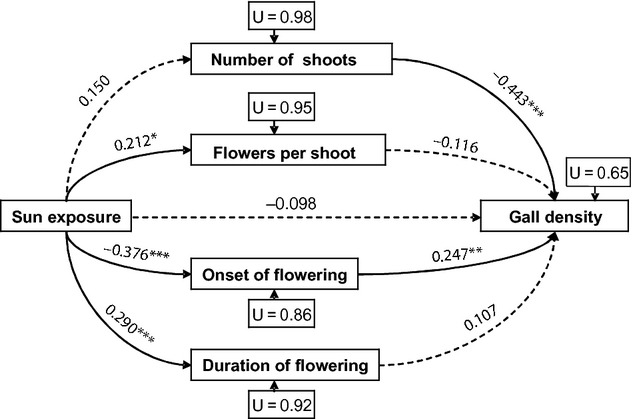
Path diagram describing the relationships between sun exposure, plant characteristics, and gall density. The analysis was based on all individuals (*n* = 139) with no missing values in any of the variables (listwise deletion). The variables gall density, number of shoots, and number of flowers per shoot were log-transformed prior to the analysis to normalize their distribution. U denotes the unexplained variance. Numbers on arrows show direct causal covariation (cf Table 3). Dashed lines indicate non-significant- and solid lines significant relationships. *P < 0.05, **P < 0.01, ***P < 0.001.

A path analysis revealed that even though sun exposure was not included in the stepwise regression model, there were indirect effects through the pattern of flowering (Fig. 6, Table 3). The more sun-exposed the plants were the earlier they started to flower, which in turn had a negative effect on gall density (Fig. 6). There is also a tendency, albeit not significant, that more sun-exposed plants have more shoots, which strongly affects gall density. Of the total effect of sun exposure on gall density, 61% was indirect through plant variables.

**Table 3:** Decomposition of bivariate covariation of gall density, plant variables, and sun exposure. The analysis was based on all individuals (*n* = 139) with no missing values in any of the variables (listwise deletion)

Relationship	Total covariation	Causal covariation Direct	Indirect	(%)	Non-causal covariation
Sun exposure – Log no of shoots	0.150	0.150	–	–	–
Sun exposure – Log (flowers/shoot)	0.212	0.212	–	–	–
Sun exposure – Onset of flowering	−0.376	−0.376	–	–	–
Sun exposure – Duration of flowering	0.289	0.289	–	–	–
Log gall density – Log no. of shoots	−0.511	−0.443	–	–	−0.068
Log gall density – Log (flowers/shoot)	−0.170	−0.116	–	–	−0.054
Log gall density – Onset of flowering	0.365	0.247	–	–	0.118
Log gall density – Duration of flowering	−0.123	0.107	–	–	−0.230
Sun exposure – Log gall density	−0.250	−0.098	−0.152	(61)	–

**Figure 7:**
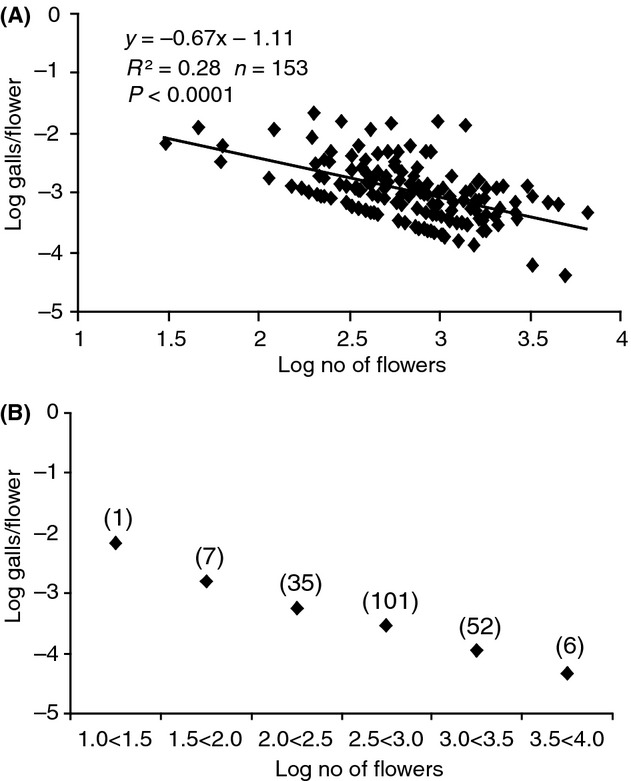
Gall density in relation to the number of flowers on individual plants. (A) Linear regression for all individuals with galls (excluding zeroes). (B) Mean gall density for all plant individuals (including those which never had any galls) arranged into size categories with regard to number of flowers (all galls divided by all flowers in each category over the 5 years). Numbers within parentheses show number of plants in each category.

An analysis of the relationship between gall density and the actual gall midge resource (flowers per individual plant) shows a strong under-exploitation of plant individuals with many flowers (Fig. 7A). Flowers on the largest plants are two orders of magnitude less attacked than those on the smallest plants. A very similar picture was shown when the analysis was performed on size classified plants (to include plants with zero galls) (Fig. 7B), confirming the strong under-exploitation of large individuals.

**Figure 8:**
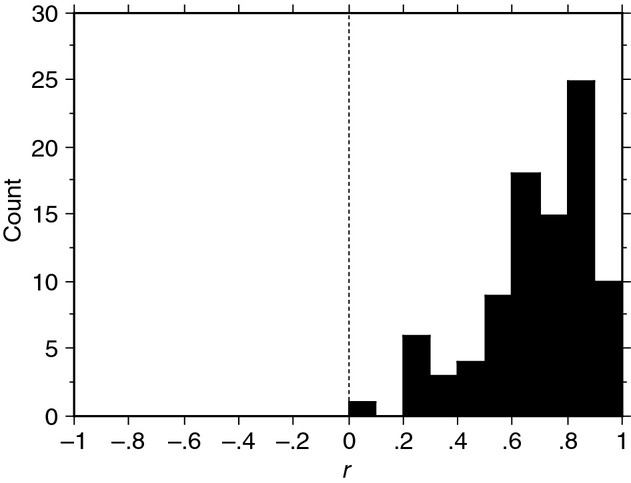
Distribution of *r* for all pairwise correlations of gall time series in the 5 × 5 m squares.

The time series for gall abundance in each 5 m square of the study area are all strongly positively correlated (Fig. 8). There is no significant spatial correlation of correlation coefficients of the pairwise correlations between all time series (Mantel statistic *r*: −0.2379, *P*: 0.9552, 9999 permutations).

## Discussion

The system studied is characterized by considerable small scale spatial variation. Plant individuals vary much in size, flower abundance and flowering phenology, and with regard to their exposure to sunshine (Fig. 3). The system also shows temporal stability; plant individuals do not change much over time with regard to number of shoots or flowers (Ågren et al. 2008). Local resources used by the gall midge are thus rather stable from year to year. These plant characteristics in combination with an insect life history characterized by an extended larval diapause (Solbreck and Widenfalk 2012), and poor adult dispersal has the potential of producing an historical imprint of insect population density and resource exploitation on the very local scale. For example, Maron and Harrison (1997) showed that a combination of poor dispersal and strong enemy interactions could produce within-habitat patterns in a tussock moth. There is also the possibility that different parts of the study area show asynchronous changes in density. However, our analysis of the spatial correlation of gall population change did not reveal any such spatial patterns. The temporal fluctuations in gall density in different parts of the study area were strongly synchronized.

Due to the very long life cycle in *C. vincetoxici* (Solbreck and Widenfalk 2012), we analyze average resource exploitation over a 5-year period, which presumably represents much of an insect cohort that emerges over several years. The vast majority of plant individuals had galls during the study period although only a very small proportion of flowers were ever used (<1%). This and similar observations from other areas (Widenfalk & Solbreck unpubl. data) give no indications for the existence of resistant plant individuals. The low attack rate by the gall midge in combination with an equally low proportion of flowers producing fruit and seed (Ågren et al. 2008) suggests that the effect of the gall midge on host plant reproduction (and thus probably also on plant fitness) is very small. Parasitoids probably play an important role in keeping gall midge populations at low average levels (Widenfalk 2002).

Two factors are identified, which are directly correlated with gall density (proportion of flowers galled). The strongest effect is from plant size (expressed as number of shoots); large plant individuals are much less efficiently used than small ones. This relationship is present even if gall density is analyzed for each year separately (not shown). Negative relationships between insect density and plant or patch size have been shown for several insect herbivores (Kareiva 1983; Bach 1988; Förare and Engqvist 1996; Förare and Solbreck 1997; Otway et al. 2005; Singer and Wee 2005; Hambäck et al. 2010), the underlying mechanisms can, however, vary. For *C. vincetoxici*, a possible explanation relates to the fact that *V. hirundinaria* individuals form fairly discrete objects (Fig. 1). If ovipositing females search for discrete host plant objects rather than for separate flowers, gall density would decrease with plant size. However, mechanisms of patch exploitation by insects are multi-faceted and other mechanisms are possible.

There is also a moderate effect of phenology; gall density is higher in plants which start flowering later (Table 2, Fig. 5B). This suggests that the timing between gall midge and host plant development is important as in many other phytophagous insects (Hunter 1992; Ngakan and Yukawa 1997; Yukawa 2000; Rehill and Schultz 2002; Albrectsen et al. 2008). *Contarinia vincetoxici* females lay their eggs in the very small flower buds soon after they have become visible. However, *V. hirundinaria* individuals produce flowers over an extended period of time, suggesting that synchrony with the earliest flowering host plants is not essential. If the emergence of the first females is somewhat later than the emergence of the first flower buds, then early flowering plants will be less exploited than later flowering ones. It is possible that early flowering individuals are more subjected to drought because they grow in more sun-exposed positions. Even though this may not manifest itself very often, late female emergence may be adaptive.

Interactions with abiotic site conditions and/or trophic web interactions may have strong effects on patterns of resource exploitation by insects. For example, enemies may distribute themselves in relation to resource densities and strongly modify the direct responses to food resources (e.g., Zeipel et al. 2006), or site characteristics may modify plant growth form or phenology (Ågren et al. 2008; Albrectsen et al. 2008). We found that gall density was significantly correlated with sun exposure (Table 1). However, in a stepwise regression, no significant effect of sun exposure on gall density remained, nor did the path analysis reveal any significant *direct* effects. However, there were significant *indirect* effects via the onset of flowering; sun-exposed plants flower earlier and are less attacked (Fig. 6).

In conclusion, the most important pattern in this study is the considerable under-exploitation of large host plant individuals compared with small ones. Although other variables, especially flowering phenology and sun exposure either directly or indirectly affect the density of galls, these effects are generally small compared with the effects of plant size.
